# Supplementary material for: The model alga Chlamydomonas reinhardtii forms mutualistic interactions with Verticillium fungi
Source: ISME Commun. 2026 Oct 6;6(1):ycag283. doi: 10.1093/ismeco/ycag283 (PMC13704775; doi:10.1093/ismeco/ycag283)
Supplement: Supplementary file 1 [file supplementary_information_final_ycag283.pdf]

Supplementary Material

The model alga *Chlamydomonas reinhardtii* forms mutualistic interactions with *Verticillium* fungi

Zoe Prockl[1], Urs Neubacher[1], Yukiyo Sato[1,$] and Hanna Rovenich[1,*]

Affiliations

[1]Institute for Plant Sciences, University of Cologne, Germany

[$]Current address: Institute of Plant Science and Resources, Okayama University, Kurashiki, Okayama 710-0046, Japan

*Correspondence to: hanna.roevenich@uni-koeln.de

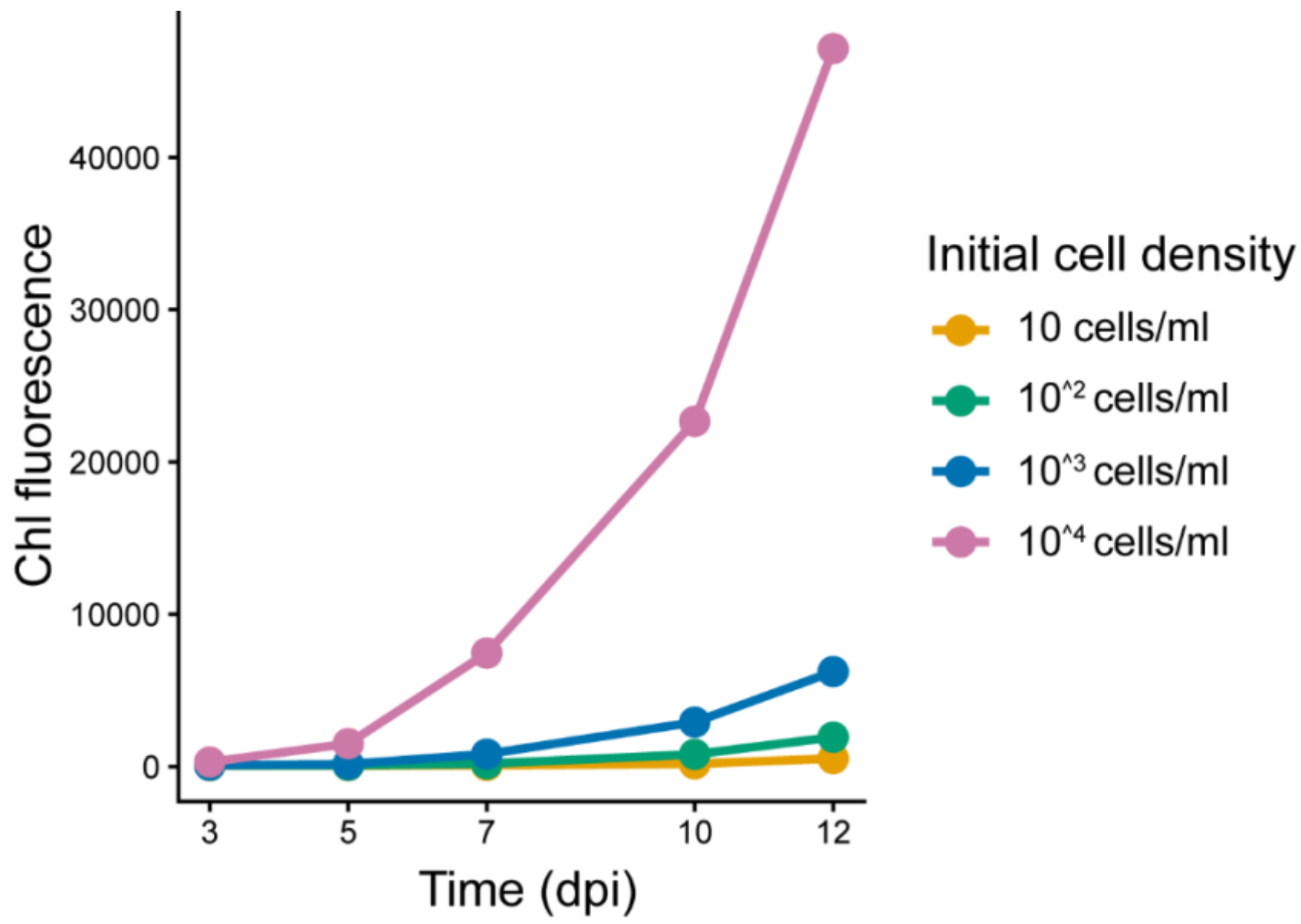

Supplementary Figure S1. Chlorophyll fluorescence correlates with algal cell density. Axenic *C. reinhardtii* cultures were inoculated at increasing initial cell densities and monitored over time. Higher starting densities resulted in proportionally higher fluorescence signals, supporting the use of chlorophyll fluorescence as a proxy for algal biomass.

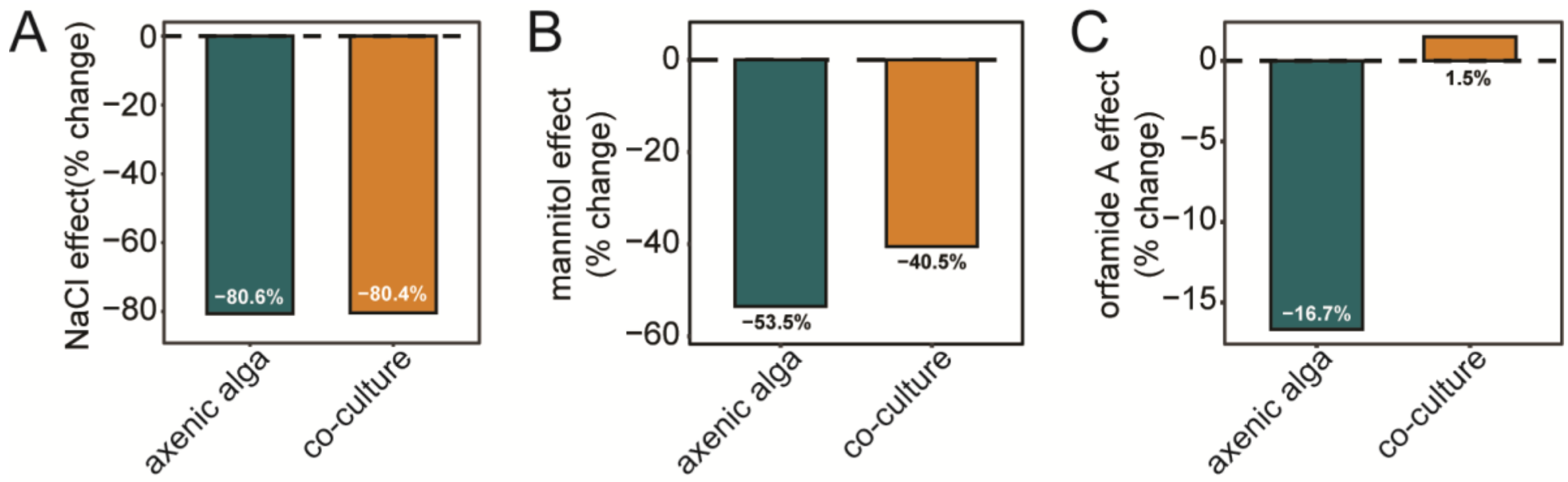

Supplementary Figure S2. Effect sizes of stress treatments in axenic and co-culture conditions. Percent change in chlorophyll fluorescence under NaCl (A), mannitol (B), and orfamide A (C) treatment relative to untreated controls. Values indicate the magnitude of stress-induced fluorescence intensity reduction in axenic *C. reinhardtii* cultures and in co-culture with *V. dahliae* from 3 independently performed experiments for NaCl and mannitol treatments, and from two independent experiments comprising four experimental units for orfamide A.
